# Primary-tiller panicle number is critical to achieving high grain yields in machine-transplanted hybrid rice

**DOI:** 10.1038/s41598-020-59751-4

**Published:** 2020-02-18

**Authors:** Min Huang, Shuanglü Shan, Jialin Cao, Shengliang Fang, Alin Tian, Yu Liu, Fangbo Cao, Xiaohong Yin, Yingbin Zou

**Affiliations:** 1 Crop and Environment Research Center, College of Agronomy, Hunan Agricultural University, Changsha, 410128 China; 2 Guangxi Key Laboratory of Rice Genetics and Breeding, Rice Research Institute, Guangxi Academy of Agricultural Sciences, Nanning, 530007 China

**Keywords:** Ecophysiology, Plant sciences

## Abstract

The development of machine-transplanted hybrid rice is a feasible approach to meet the needs of both high grain yield and high labor efficiency in China, but limited information is available on the critical plant traits associated with high grain yields in machine-transplanted hybrid rice. This study was carried out to identify which type of culms (*i.e*., main stems and primary and secondary tillers) and which yield components of this culm are critical to achieving high grain yields in machine-transplanted hybrid rice. Field experiments were conducted with two hybrid rice cultivars grown under two densities of machine transplanting in two years. Results showed that total grain yield of main stems and primary and secondary tillers was not significantly affected by cultivar but was significantly affected by density and year. Averaged across cultivars, densities, and years, main stems and primary and secondary tillers contributed about 15%, 50%, and 35% to total grain yield, respectively. Total grain yield was not significantly related to grain yields of main stems and secondary tillers but was positively and significantly related to grain yield of primary tillers. Approximately 85% of the variation in total grain yield was explained by grain yield of primary tillers, which was positively and significantly related to primary-tiller panicles per m^2^ but not to spikelets per panicle, spikelet filling percentage, or grain weight of primary tillers. Based on these results, it is concluded that primary-tiller panicle number is essential for achieving high grain yields in machine-transplanted hybrid rice.

## Introduction

Rice is the staple food crop for more than 65% of the population in China^1^, and rice self-sufficiency has been achieved in China^2^. However, changes in the Chinese socio-economic environment in recent years have posed challenges for rice production. A massive rural-to-urban migration in China has led to decreased land and labor resources and increased labor wages for rice production^2,3^. Therefore, improving the grain yield per unit land area with less labor inputs is crucial for maintaining rice self-sufficiency in China.

The development of hybrid rice has the potential to increase the grain yield by 10–20%, and China has successfully exploited the potentials of hybrid rice on a large scale^4,5^. In addition, machine transplanting is a labor-saving crop establishment method that has been developed rapidly for rice production in China^6^. Taken together, developing machine-transplanted hybrid rice should be a feasible approach to meet the needs of both high grain yield and high labor efficiency in China.

The grain yield of rice includes grain yields of different culms (*i.e*., main stems and different tillers), which are determined by panicles per m^2^, spikelets per panicle, spikelet-filling percentage, and grain weight. There have been reports that the yield components are different among main stems and different tillers in rice and are dependent on cultivar traits, agronomic practices (*e.g*., planting density and N application rate), and environmental conditions^7–9^. However, these previous studies were conducted under manually transplanted or direct-seeded conditions, and limited information is available on the yield components of main stems and different tillers and their relationships to grain yield in machine-transplanted rice. It has been well-documented that the yield components of rice can be influenced by crop establishment method^10,11^, so it is necessary to study yield under all establishment methods.

In the present study, grain yield and yield components of different culms (*i.e*., main stems and primary and secondary tillers) were determined in two hybrid rice cultivars grown under two densities of machine transplanting in two years. The objective of this study was to identify which type of culms and which yield components of this culm are critical to achieving high grain yields in machine-transplanted hybrid rice.

## Results

There was no significant difference in total grain yield between L1212 and T390 (Table 1). The difference in grain yield of main stems was also not significant between the two cultivars. L1212 produced 9% higher grain yield of primary tillers but 20% lower grain yield of secondary tillers than T390.

**Table 1:** Total grain yield and grain yield of main stems and primary and secondary tillers in two hybrid rice cultivars grown under two densities in 2017 and 2018.

Variable	Grain yield (g m^−2^)			
	Total	Main stems	Primary tillers	Secondary tillers
**Cultivar (C)**^**a**^				
L1212	722 a	105 (15) a	391 (54) a	227 (31) b
T390	756 a	115 (15) a	359 (48) b	282 (37) a
**Density (D)**^**b**^				
D11	836 a	137 (17) a	453 (54) a	246 (29) a
D21	642 b	82 (13) b	296 (46) b	264 (41) a
**Year (Y)**				
2017	647 b	117 (18) a	335 (52) b	195 (30) b
2018	831 a	102 (12) b	414 (50) a	315 (38) a
**Interaction**				
C × D	ns	ns	ns	ns
C × Y	ns	ns	ns	ns
D × Y	**	ns	**	**
C × D × Y	*	ns	**	ns

^a^L1212 and T390 are Longjingyou 1212 and Taiyou 390, respectively.

^b^D11 and D21 represent a hill spacing of 25 cm × 11 cm and 25 cm × 21 cm, respectively.

Values in parentheses are percentages of total grain yield.

Means within each variable sharing the same letter are not significantly different at the 0.05 probability level.

ns denotes non-significance at the 0.05 probability level; * and ** denote significance at the 0.05 and 0.01 probability levels, respectively.

D11 produced 30% higher total grain yield than D21 (Table 1). Grain yields of main stems and primary tillers were 67% and 53% higher under D11 than under D21, respectively. There was no significant difference in grain yield of secondary tillers between the two densities.

Total grain yield was 22% lower in 2017 than in 2018 (Table 1). Grain yield of main stems in 2017 was 15% higher than that in 2018. Grain yields of primary and secondary tillers were lower in 2017 than in 2018 by 19% and 38%, respectively.

There were no significant interactive effects of C × D or C × Y on total grain yield and grain yields of main stems and primary and secondary tillers (Table 1). The interactive effect of D × Y was significant for total grain yield and grain yields of primary and secondary tillers but not significant for grain yield of main stems. The interactive effect of C × D × Y was significant for total grain yield and grain yield of primary tillers but not significant for grain yields of main stems and secondary tillers.

**Figure 1:**
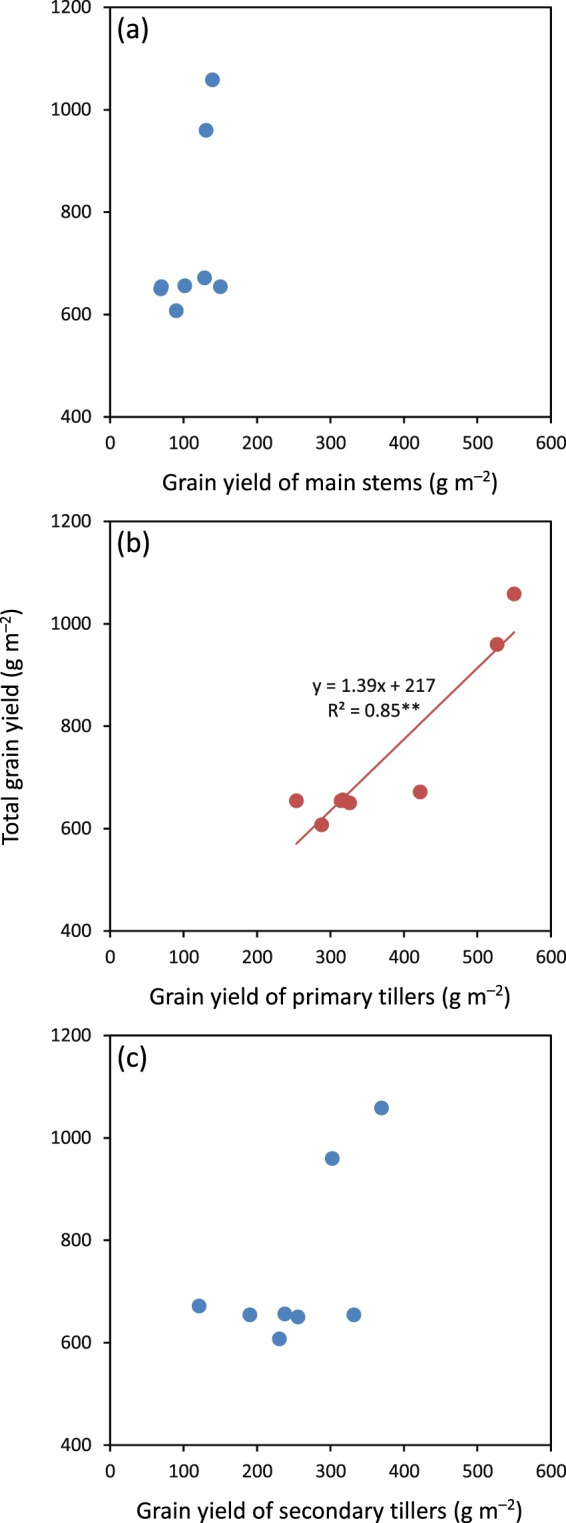
Relationships between total grain yield with grain yield of main stems (**a**), primary tillers (**b**), and secondary tillers (**c**) in hybrid rice. ** denotes significance at the 0.01 probability level.

Averaged across cultivars, densities, and years, main stems and primary and secondary tillers contributed about 15%, 50%, and 35% to total grain yield, respectively (Table 1). Total grain yield was not significantly related to grain yields of main stems and secondary tillers but positively and significantly related to grain yield of primary tillers (Fig. 1a–c). Approximately 85% of the variation in total grain yield was explained by grain yield of primary tillers (Fig. 1b). As a consequence, only the yield components of primary tillers are included in the following analysis.

There were no significant differences in panicles per m^2^, spikelets per panicle, or grain weight of primary tillers between L1212 and T390 (Table 2). Spikelet filling percentage of primary tillers was about 8% higher in L1212 than in T390.

**Table 2:** Yield components of primary tillers in two hybrid rice cultivars grown under two densities in 2017 and 2018.

Variable	Panicles m^−2^	Spikelets panicle^−1^	Spikelet filling (%)	Grain weight (mg)
**Cultivar (C)**^**a**^				
L1212	132 a	192 a	73.2 a	21.4 a
T390	126 a	196 a	65.6 b	22.1 a
**Density (D)**^**b**^				
D11	159 a	187 a	70.0 a	21.8 a
D21	99 b	201 a	68.9 a	21.8 a
**Year (Y)**				
2017	121 b	182 b	71.4 a	22.1 a
2018	137 a	206 a	67.5 b	21.5 a
**Interaction**				
C × D	ns	ns	**	ns
C × Y	ns	ns	*	ns
D × Y	ns	**	*	ns
C × D × Y	ns	ns	*	ns

^a^L1212 and T390 are Longjingyou 1212 and Taiyou 390, respectively.

^b^D11 and D21 represent a hill spacing of 25 cm × 11 cm and 25 cm × 21 cm, respectively.

Means within each variable sharing the same letter are not significantly different at the 0.05 probability level.

ns denotes non-significance at the 0.05 probability level; * and ** denote significance at the 0.05 and 0.01 probability levels, respectively.

D11 had 61% higher primary-tiller panicles per m^2^ than D21 (Table 2). The differences in spikelets per panicle, spikelet filling percentage, and grain weight of primary tillers were not significant between the two densities.

Both panicles per m^2^ and spikelets per panicle of primary tillers were 12% lower in 2017 than in 2018 (Table 2). Spikelet filling percentage of primary tillers was about 4% higher in 2017 than in 2018. There was no significant difference in grain weight of primary tillers between the two years.

The interactive effects of C × D, C × Y, and C × D × Y were not significant for panicles per m^2^, spikelets per panicle, or grain weight of primary tillers but were significant for spikelet filling percentage of primary tillers (Table 2). The interactive effects of D × Y were not significant for panicles per m^2^ or grain weight of primary tillers but were significant for spikelets per panicle and spikelet filling percentage of primary tillers.

**Figure 2:**
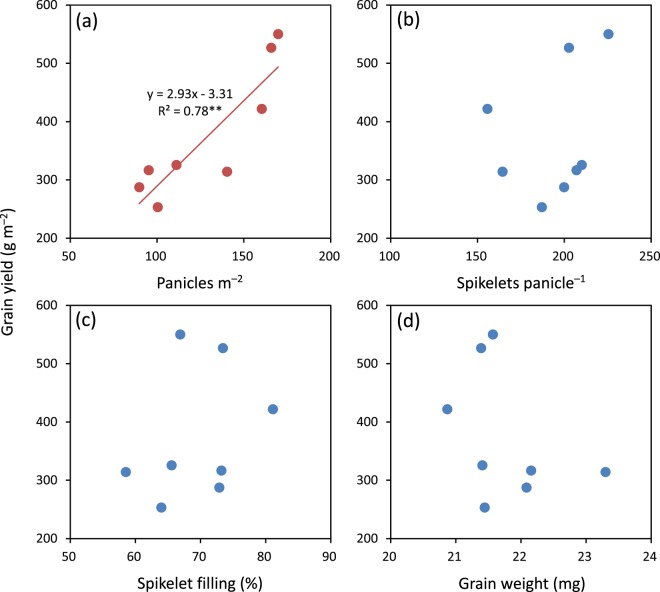
Relationships between grain yield with panicles per m^2^ (**a**), spikelets per panicle (**b**), spikelet filling percentage (**c**), and grain weight (**d**) of primary tillers in hybrid rice. ** denotes significance at the 0.01 probability level.

There was a significant and positive relationship between grain yield of primary tillers and primary-tiller panicles per m^2^ (Fig. 2a). Grain yield was not significantly related to spikelets per panicle, spikelet filling percentage, or grain weight of primary tillers (Fig. 2b–d).

## Discussion

In this study, total grain yield was not significantly affected by cultivar. This result is consistent with our previous studies^12,13^, which used the same two cultivars (L1212 and T390) as the present study and showed that the two cultivars produced similar grain yields. These results indicate that the two cultivars have a comparable yield potential.

Although this study did not find a significant cultivar difference in total grain yield, a significant effect of hill density on total grain yield was observed. Namely, a 30% increase in total grain yield was achieved by increasing hill density from 25 cm × 21 cm to 25 cm × 11 cm. This result indicates that increasing hill density is a feasible way to increase grain yield in machine-transplanted hybrid rice. This finding is in agreement with that reported in hybrid rice grown under manually transplanted conditions^14^. The increased grain yield with increasing hill density in hybrid rice may be because panicles per m^2^ is the critical yield component for achieving high grain yield in hybrid rice^15^. The results of this study further showed that the increased total grain yield from increasing hill density was mainly attributable to increased grain yields of main stems and primary tillers.

**Figure 3:**
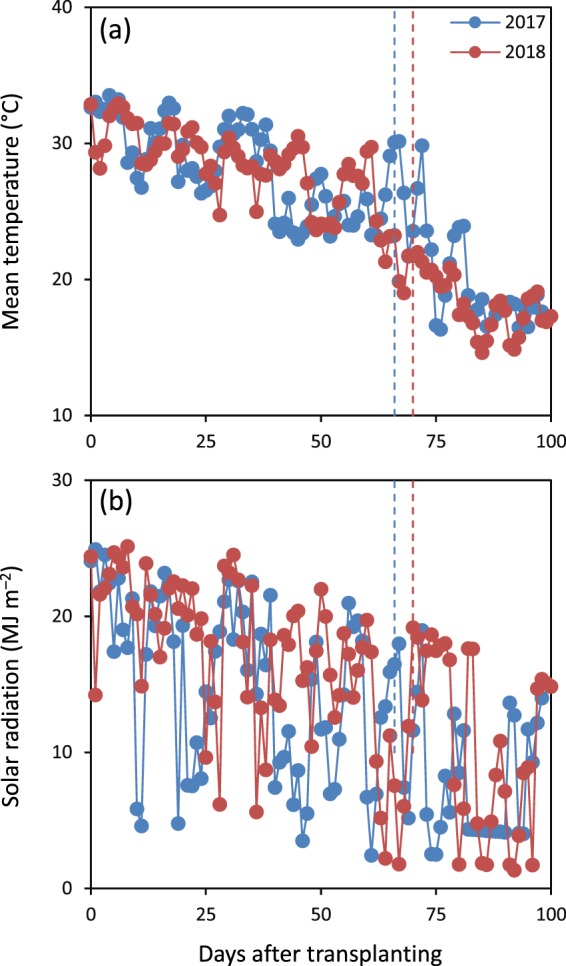
Daily solar radiation (**a**) and mean temperature (**b**) during the rice-growing season in 2017 and 2018. Vertical dashed lines represent the heading stage.

A significant yearly variation in total grain yield was also observed in this study, *i.e*., total grain yield was lower in 2017 compared to 2018. The lower total grain yield in 2017 compared to 2018 was partly due to lower grain yield of primary tillers, which was driven by lower panicles per m^2^ and spikelets per panicle of primary tillers. The yearly differences in panicles per m^2^ and spikelets per panicle of primary tillers could be explained by the yearly variation in accumulative solar radiation during the pre-heading period, which was 16% lower in 2017 than in 2018 (Fig. 3a). In addition, lower grain yield of secondary tillers was also partially responsible for the lower total grain yield in 2017 than in 2018. The difference in grain yield of secondary tillers was mainly attributable to variation in secondary-tiller panicles per m^2^ (89 in 2017 *vs*. 150 in 2018) (data not shown) caused by change in accumulative solar radiation during the pre-heading period. Low light intensity can significantly reduce the tiller number due to lack of photosynthates^16^.

Some significant interactive effects of the experimental factors (cultivar, density, and year) on yield attributes were also found in this study. Spikelet filling percentage of primary tillers was significantly affected by all two-factor and three-factor interactions. This result also highlights why spikelet filling percentage is the yield component that is difficult to control or regulate in rice production.

In addition, and most importantly, the comprehensive analysis of data across cultivar, density, and year in this study provided useful quantified information for identifying critical plant traits associated with high grain yields in machine-transplanted hybrid rice, including: (1) primary tillers contributed about 50% of total grain yield, followed by secondary tillers (35%) and main stems (15%); and (2) 85% of variation in total grain yield was explained by grain yield of primary tillers, which was closely related to primary-tiller panicles per m^2^. Accordingly, we can conclude that panicle number of primary-tillers is essential in achieving high grain yields in machine-transplanted hybrid rice. However, there is a limitation in the present study that needs to be acknowledged, namely, only two hill densities with a single seedling per hill were employed in this study. Therefore, further investigations involving more hill densities and seedling number per hill should be done to confirm the conclusion of this study.

## Methods

Field experiments were conducted in Yongan (28°09′N, 113°37′E, 43 m asl), Hunan Province, China in the late rice-growing season in 2017 and 2018. The site has a moist subtropical monsoon climate. Average daily solar radiation and mean temperature during the rice-growing season were 12.9 MJ m^−2^ d^−1^ and 25.7 °C in 2017 and 15.1 MJ m^−2^ d^−1^ and 25.0 °C in 2018, respectively (Fig. 3a,b). The soil of the experimental field was a clay with pH 6.20, organic matter 38.6 g kg^−1^, available N 168 mg kg^−1^, available P 18.5 mg kg^−1^, and available K 183 mg kg^−1^. Soil tests were based on samples taken from the upper 20 cm layer before the experiment started in 2017.

In each year, two hybrid rice cultivars, Longjingyou 1212 (L1212) and Taiyou 390 (T390), were grown under two hill densities, 25 cm × 11 cm (D11) and 25 cm × 21 cm (D21). The cultivars were selected because they are commonly grown in the study region. The experiment was laid out in a split-plot design using hill densities as main plots and cultivars as subplots, with three replications and a subplot size of 80 m^2^.

Rice seedlings were raised according to the method described by Huang and Zou^17^, and 27-day-old seedlings were transplanted with one seedling per hill, using a high-speed rice transplanter (PZ80-25, Dongfeng Iseki Agricultural Machinery Co., Ltd., Xiangyang, China). Missing plants were manually replanted within 7 days after transplanting to obtain a uniform plant population. Fertilizers used were urea for N, single superphosphate for P, and potassium chloride for K at rates of 150 kg N ha^−1^, 75 kg P_2_O_5_ ha^−1^, and 150 kg K_2_O ha^−1^, respectively. The N fertilizer was applied in three splits: 50% as basal fertilizer (1 d before transplanting), 20% at early-tillering (7 days after transplanting), and 30% at panicle initiation. The P fertilizer was applied as basal fertilizer. The K fertilizer was split equally at basal fertilization and panicle initiation. The experimental field was kept flooded with a water depth of 3–5 cm from transplanting until 7 days before maturity. Weeds, pests, and diseases were intensively controlled by chemicals to avoid yield loss.

Ten hills of rice plants were sampled from each subplot and separated into three subsamples: main stems, primary tillers, and secondary tillers. The main stem is the first plant stem. Primary and secondary tillers are those that emerged from the main stem and primary tillers, respectively. Panicle number was counted to calculate panicles per hill and per m^2^ for each subsample. The panicles were hand threshed, and filled and unfilled spikelets were counted to calculate spikelets per panicle and spikelet filling percentage. Dry weight of filled grains was determined after oven-drying at 70 °C to a constant weight, and grain weight was calculated. Total grain yield was the summation of grain yields (filled grain weights) of main stems, primary tillers, and secondary tillers.

Data were analysed using analysis of variance (ANOVA) and linear regression (Statistix 8.0, Analytical Software, Tallahassee, FL, USA). The statistical model for the ANOVA included replication, cultivar (C), density (D), year (Y), the two-factor interactions of C × D, C × Y, and D × Y, and the three-factor interaction of C × D × Y. Statistical significance was set at the 0.05 probability level.

## Data Availability

All data generated or analysed during this study are included in the article.
